# Clinical Overview of GIST and Its Latest Management by Endoscopic Resection in Upper GI: A Literature Review

**DOI:** 10.1155/2018/6864256

**Published:** 2018-10-31

**Authors:** Cicilia Marcella, Rui Hua Shi, Shakeel Sarwar

**Affiliations:** ^1^Department of Gastroenterology, Southeast University Affiliated Zhongda Hospital, Nanjing 210009, China; ^2^Department of Orthopedics, Southeast University Affiliated Zhongda Hospital, Nanjing 210009, China

## Abstract

**Aims:**

To review the clinical presentation, diagnosis, assessment of risk of malignancy, and recent advances in management (mainly focusing on the role of endoscopic resection) of gastrointestinal stromal tumors (GISTs) in upper GI.

**Method:**

We searched Embase, Web of science, and PubMed databases from 1993 to 2018 by using the following keywords: “gastrointestinal stromal tumors,” “GIST,” “treatment,” and “diagnosis.” Additional papers were searched manually from references of the related articles.

**Findings:**

The improvement of endoscopic techniques in treating upper gastrointestinal subepithelial tumors especially gastrointestinal tumors has reduced the need for invasive surgery in patients unfit for surgery. Many studies have concluded that modified endoscopic treatments are effective and safe. These treatments permit minimal tissue resection, better dissection control, and high rates of en bloc resection with an acceptable rate of complications.

## 1. Introduction

Gastrointestinal stromal tumors (GISTs) are the most common mesenchymal subepithelial tumor (SET). They occur in the stomach (60–70%), small intestine (20–30%), duodenum (4-5%), rectum (4-5%), colon (<2%), and esophagus (<1%) [1–3]. They are rarely found in the peritoneum, mesentery, and omentum [4]. GISTs have been proved to arise from the smooth muscle pacemaker interstitial cell of Cajal (ICC) which has a function of coordinating gut motility [5] and peristalsis. GISTs demonstrate a higher incidence rate in men and among blacks, and most patients are between 40 and 80 years old at the time of diagnosis, with a median age of 63 years [6].

Prompt treatment of upper GISTs is very crucial. According to the latest guidelines of NCCN, ESMO, and Japan, a GIST less than 2 cm with no signs of malignancy may be managed with active surveillance. A small tumor size does not exclude the malignant potential in a GIST. Thus, despite the size, the patient should be told about the possibility of malignancy. Many studies have proved the feasibility and safety of endoscopic approaches in treating upper GISTs. These procedures include endoscopic band ligation (EBL), endoscopic submucosal excavation (ESE), endoscopic submucosal dissection (ESD), endoscopic mucosal dissection (EMD), endoscopic submucosal tunnel dissection (ESTD), submucosal tunneling endoscopic resection (STER), endoscopic full-thickness resection (EFTR), laparoscopic endoscopic cooperative surgery (LECS), nonexposed endoscopic wall-inversion surgery (NEWS), and a combination of laparoscopic and endoscopic approaches to neoplasia with a nonexposed technique (CLEAN-NET). We will discuss all the above procedures in this review along with their respective steps. We will also discuss the clinical presentation, malignant potential, and diagnosis of GISTs through imaging and pathology.

## 2. Clinical Presentation, Imaging, and Pathological Diagnosis

The symptoms of GISTs are nonspecific and depend on the size and location [7]. Many small GISTs (<2 cm) are usually found parenthetically by endoscopy or imaging, since many of them show no symptoms [8]. The most common symptom is gastrointestinal (GI) bleeding, which is present in approximately 50% of the patients, followed by abdominal pain (20–50%) and GI obstruction (10–30%). Other symptoms include melena, hematemesis, fullness, and palpable mass. GISTs that are located in the proximal stomach may lead to dysphagia, while tumors located in the pylorus may present as gastric outlet obstruction [9, 10]. GISTs can be a part of a syndrome called Carney's triad (gastric GIST, pulmonary chondroma, and paraganglioma) or neurofibromatosis type 1 (mostly spindle cell GIST) [11]. GISTs frequently metastasize to the liver and rarely spread to the regional lymph node or other extra-abdominal organs [12].

**Figure 1:**
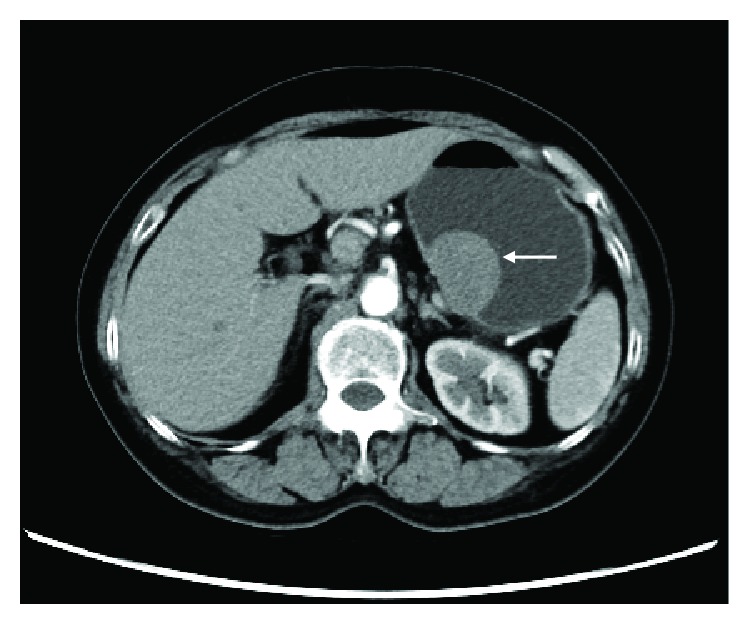
An approximately 3.9^∗^2.8 cm gastrointestinal tumor on the lesser curvature of the stomach body seen on enhanced CT imaging (white arrow).

An initial investigation should include a detailed history and thorough physical examination, followed by imaging studies to both assess the extent of the primary tumor and evaluate the presence of metastatic disease. According to the latest NCCN guidelines, a CT (computed tomography) scan of the abdomen/pelvis is the initial workup for the evaluation, staging, and monitoring of treatment response in a GIST. GISTs typically showed a well-defined soft tissue of relatively low density, which is homogenous on a contrast-enhanced CT scan (Figure 1). On MRI, GISTs typically showed a well-defined, low to intermediate signal intensity on T1-weighted images and high signal intensity on T2-weighted images.

**Figure 2:**
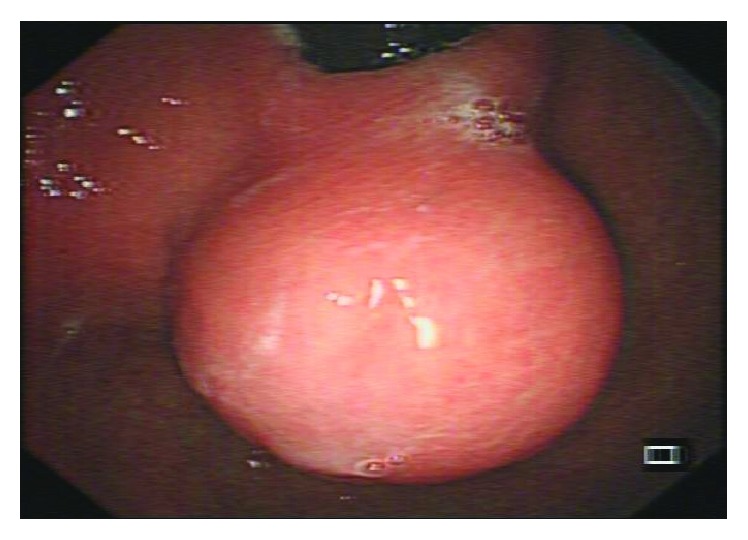
A large gastrointestinal tumor located in the lower part of the cardia seen under endoscopy forming a smooth contoured elevation.

GISTs under endoscopic procedure typically form a well-delineated spherical or hemispheric mass, arising mostly from the muscularis propria (MP) layer beneath the mucosa and pushing it to the lumen to form a smooth contoured elevation (Figure 2). GISTs are usually well circumscribed and surrounded by a pseudocapsule which contributes to the indications for complete resection in endoscopic enucleation.

The pathological diagnosis of a GIST is determined by morphology and immunohistochemical (IHC) findings. The most important one is KIT (CD117), a tyrosine kinase inhibitor which is a transmembrane protein that stimulates cell proliferation and inhibits apoptosis. It presents in almost 95% of GISTs [13]. CD34 expression was also considered to be the most valuable marker before the recognition of the CD117 antibody, and it presents in between 40% and 82% of GISTs [14]. Thus, CD34 expression was accepted as a diagnostic supportive “marker” until now. CD117 can help in distinguishing GISTs from other gastrointestinal mesenchymal tumors, since it is not expressed in smooth muscle or neural tumors [15]. However, some may show CD117 negative, typically the PDGFR*α* (platelet-derived growth factor *α*) mutant or wild types. Thus, DOG1 is added as an alternative marker as a supplement in diagnosing GISTs [16]. The 3 main morphological types of GISTs include spindle cell type (70%), epithelioid cell type (20%), and mixed type (10%), which is highly malignant.

## 3. Malignant Potential

**Figure 3:**
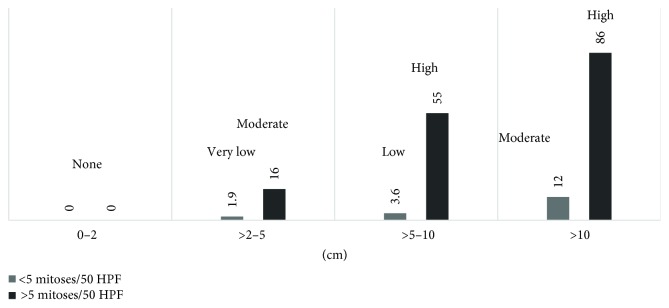
Gastric GISTs: risk assessment of malignant potential.

Assessing the malignant potential in GIST patients is crucial for deciding the next step in treatment. The prognosis of a GIST is highly associated with mitotic count, tumor size, tumor necrosis, anatomical location, invasive growth, and expression of Ki-67 and PCNA index [17, 18]. Tumors with a size greater than 10 cm showing calcifications, irregular margins, heterogeneity, lobulation, and ulceration, along with extraluminal and mesenteric fat infiltration, are more likely to be associated with metastasis [19]. The chart in Figure 3 shows the gastric predictors in assessing the malignant potential of a GIST, according to the latest national comprehensive cancer network (NCCN) guidelines. As shown in the chart, the vertical axis stands for the metastatic rate (%) and the horizontal axis stands for the tumor size (cm) as well as the 2 series for mitotic rate (/50 HPFs). Gastric GISTs with a size of ≤10 cm and having ≤5 mitoses per 50 HPFs have a low malignancy potential [2]. Overall, tumors < 5 cm, and especially <2 cm, have a lower risk of metastasis, in contrast to tumors >5 cm, and especially >10 cm, which have a higher risk of metastasis. For the mitotic rate of <5 mitoses/50 HPF, there is a lower risk of metastasis, compared to those tumors with mitotic rates > 5/50 HPF. Mitotic rates > 10/50 HPF indicate a higher risk of metastasis [20]. These two factors are independent but mutually influential predictors, and are thus added in the NIH guidelines. However, the diagnosis and prediction of the malignant potential of GIST are still difficult.

## 4. Role of Endoscopy in GIST Patient

Endoscopy has been used worldwide for many purposes. The widespread application of endoscopy and endoscopic ultrasound (EUS) has led to the detection of many early-stage upper GISTs, giving a chance of complete resection. Many authors have claimed that EUS is the most appropriate method for esophagogastric submucosa tumors. A GIST on EUS will appear as hypoechoic, inhomogeneous, anechoic, or having a high echo (when tumors are malignant), and it is commonly located in the third and fourth layer, and rarely in the second layer [21]. EUS may also be used for the prediction of malignancy as well [15]. Palazzo et al. [22] concluded that EUS features suggestive of malignancy include enlarged lymph nodes, size greater than 4 cm, irregular margins, and the presence of cystic spaces within the mass. For a tumor of larger size, EUS can be very useful in differentiating a submucosal tumor (SMT) from extrinsic compression, with 92% sensitivity and 100% specificity [23].

According to their location in the gastric wall, GISTs are classified into 4 types: type 1 (very narrow connection with the MP layer which protrudes into the lumen), type 2 (wide connection with the MP which protrudes into the lumen at an obtuse angle), type 3 (located in the middle of the gastric wall), and type 4 (protrudes into the serosal side of the gastric wall) [24]. Endoscopic enucleation is best suitable for types 1 and 2. Endoscopic enucleation include EBL, ESD, EMD, ESTD, and STER. Types 3 and 4 are commonly resected by other techniques such as EFTR and more advanced methods of endoscopic and laparoscopic combination techniques, such as LECS, NEWS, and CLEAN-NET. The summaries of the included studies reporting relevant outcomes are shown in Tables 1 and 2.

### 4.1. Endoscopic Band Ligation

EBL was first applied for treating esophageal varices [25]. Later on, it was applied for treating gastrointestinal superficial lesions. For the very first time, Sun et al. [26] concluded that EBL was an effective and safe method for treating small GISTs. 96.6% (28/29) of the cases were resected completely, with a low complication rate (3.4%, 1/29) and recurrence rate (3.4%, 1/29). In this procedure, the tumor was first aspirated with a transparent cap and then ligated with the band. EUS was used to confirm that the hypoechoic mass had been completely confined by the band. The overlying mucosa and submucosal layer were then cut, thus dissecting the tumor. Many authors have demonstrated the safety and efficacy of EBL for gastric GIST [27, 28]. The hurdles of EBL are the limited size of the tumor (≤12 mm) that can be resected due to the size of the transparent cap, and EBL is suitable only for GISTs located in the superficial MP layer [29]. However, EBL is rarely used now to treat GISTs.

### 4.2. Endoscopic Submucosal Dissection

**Figure 4:**
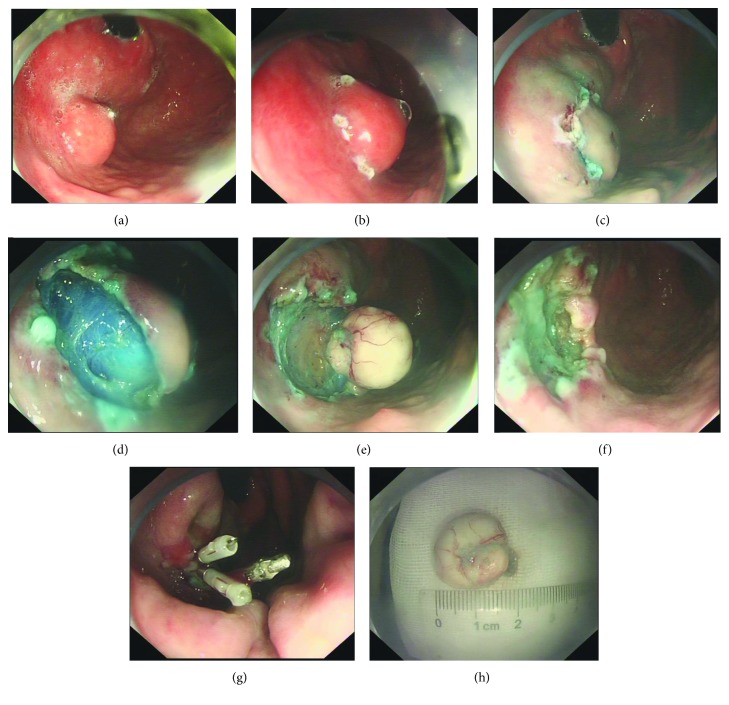
Endoscopic submucosal dissection. (a) A 2^∗^2 cm subepithelial tumor located in the gastric fundus. (b) Marking the lesion boundaries. (c) Incision of the tumor was made after lifting the submucosa layer by injecting a mixed solution into the submucosa layer. (d–f) Tumor is resected. (g) Endoscopic clips were used to close the wound. (h) The resected specimen.

ESD has been used to remove an SMT, including a GIST. The ESD standard procedure is as follows: identifying and marking the lesion boundaries, injecting a solution (a mixture of normal saline, epinephrine, and indigo carmine dye) into the submucosal layer, initial incision of the mucosa and submucosa layer, and dissecting the tumor (Figure 4). ESD allows a larger resectable size and a higher en bloc resection rate when compared with EBL. He et al. [30] demonstrated that ESD is effective, safe, and feasible in treating large-sized GISTs. A total of 31 patients underwent an ESD for larger-sized GISTs (mean size 2.7 ± 0.72 cm). The results showed favorable outcomes, although 6 patients had intraoperative perforations and were successfully managed endoscopically, with no further surgery required.

Many studies have also demonstrated that ESD is safe and effective when compared to conventional surgical approaches (open or laparoscopic). Soh et al. [31] retrospectively analyzed the comparison of ESD (55 patients) and surgery (27patients) in treating gastric subepithelial tumors (SETs). This proved that ESD is an efficient treatment for gastric SETs with the advantages of shorter hospital stays and lower hospital costs when compared with surgery. Meng et al. [32] evaluated a total of 115 SMT patients who underwent either an ESD (68/115) or laparoscopic wedge resection (LWR) (47/115). Results showed that for tumors < 2 cm and between 2 and 5 cm, ESD was associated with a shorter mean operation time, less blood loss, shorter length of hospital stays and lower cost. It also concluded that ESD can achieve the same rates of en bloc resection and complete resection compared with LWR.

### 4.3. Endoscopic Muscularis Dissection

EMD was first introduced by Liu et al. [33] as a new endoscopic technique for resecting tumors originating from the MP layer. The procedure includes injecting a solution (a mixture of epinephrine and normal saline) into the submucosal layer, marking the tumor, incising the overlying mucosa to expose the tumor, dissecting the submucosa and muscular tissue around the lesion to better reveal the tumor, and dissecting the tumor. The study included 31 patients (14 = esophageal tumor, 17 = gastric tumor). It achieved 97% (30/31) of complete resections, and the perforation rate was 13% (4/31). Thus, EMD can be a treatment of choice in treating patients with upper-GI subepithelial tumors originating from the MP.

### 4.4. Endoscopic Submucosal Tunneling

Peroral endoscopic submucosal tumor resection (POET) was first developed by Inoue et al. [34] to treat esophageal or cardia subepithelial tumors. The research concluded that the procedure is feasible for selected submucosal tumors with a size of up to 4 cm. The POET procedure for resecting SETs is referred to as submucosal tunneling endoscopic resection (STER) or endoscopic submucosal tunnel dissection (ESTD). The standard procedures include injecting a solution into the submucosal layer, creating a submucosal tunnel 5 cm above the tumor, dissecting the overlying mucosa or submucosa, dissecting the tumor from the muscular layer, retrieving the specimen, and closing the entry mucosa orifice with hemostatic clips [35–38]. POET is efficient for resecting SETs located at the esophagogastric junction and in the esophagus, which is believed to be a difficult site for laparoscopic wedge resection [39]. It also possesses numerous advantages compared to other surgical procedures, including a shorter hospital stay, lower cost, perseverance of mucosal integrity, faster healing rate, and decreased risk of gastrointestinal tract leakage and consequent infection [40–42]. POET limitations include the challenge of performing the procedure in the fundus and upper greater curvature of the stomach, and lesions larger than 4 cm are difficult to retrieve perorally.

### 4.5. Endoscopic Full-Thickness Resection

Suzuki and Ikeda [43] were the first to develop an EFTR technique. Many researches have claimed that the EFTR is a technique of choice for SETs originating from the MP layer. Zhou et al. [44] and Feng et al. [45] demonstrated a successful EFTR procedure without laparoscopic assistance on 26 (16/26 were GISTs) and 48 (43/48 were GISTs) gastric SMTs, respectively. Both claimed to have a 100% complete resection rate with no complications or recurrences in follow-up. The standard procedure includes marking the lesion and injecting a solution (a mixture of normal saline, 1% indigo carmine, and epinephrine) into the submucosal layer, circumferential incision around the lesion in the MP layer, incising the serosal layer to generate active perforation, removing the tumor with its adjacent tissues by snare, and closing the perforated gastric wall with endoscopic clips and endoloop ligature (extra closing device) [46]. Schmidt et al. [47] recommended a method called “suture first, cut later”; whereby a new suturing device is used to suture beneath the tumor after the resection is performed. This method has an advantage of resecting relatively large tumors (±4 cm), regardless of their location. Kappelle et al. [48] reported an EFTR technique using a new flat-based Padlock over-the-scope (OTS) clip for tumors < 2 cm in the gastric wall (7/13) and duodenum (6/13). A total of 13 SETs (2 GISTs) were selected. From the result, the feasibility and effectiveness of achieving 100% R0 resection can be concluded, although several cases (duodenum) were complicated by (micro)perforations. Furthermore, EFTR required the creation of a pseudoperforation, which can increase the risk of intraperitoneal tumor seeding when the pseudocapsule is not intact. Thus, more studies on a larger scale are needed to standardize this technique and skilled endoscopists are required to reduce the risk of intraperitoneal infection caused by inadequate mucosal suturing.

### 4.6. Laparoscopic Endoscopic Cooperative Surgery

LECS in GISTs is a technique that was first performed by Hiki et al. [49] in 2008. This technique is believed to minimize the dissection of the normal gastric wall with minimal gastric transformation when compared with laparoscopic wedge resection (LWR). The study analyzed 7 patients (6/7 GISTs) with a median tumor size of 4.6 cm. Results showed no intraoperative or postoperative complications. Initially, the tumor location is identified by endoscopy and laparoscopy. Argon plasma coagulation (APC) is used to mark the tumor edge followed by injecting 10% glycerin into the submucosal layer. An insulated tip (IT) knife is used to incise three-fourths of the marked area of the tumor. Subsequent laparoscopic dissection of the seromuscular layer is achieved by making a pseudoperforation, and dissection is done by an ultrasonically activated device. The incision line is sealed with laparoscopic stapling devices. LECS is best suited for gastric GISTs originating from the intramural MP layer [24]. Namikawa and Hanazaki [50] concluded that full-thickness excision using the LECS method is a promising procedure in the treatment of GISTs < 5 cm, with the advantages of reduction in the resected area and lower estimated blood loss when compared to LWR.

### 4.7. Nonexposed Endoscopic Wall-Inversion Surgery

NEWS was invented in 2010 by Goto et al. [51] to avoid the inevitable intraperitoneal seeding caused by the EFTR technique. The procedure includes endoscopically marking the edge of the lesion, laparoscopically marking the serosal side opposite the mucosal marking, endoscopically injecting a hyaluronate solution into the submucosal layer, laparoscopically incising the circumferential seromuscular layer, pushing and inverting the dissected lesion into the lumen, laparoscopically suturing the seromuscular defect, and finally achieving complete resection by ESD around the lesion. With NEWS, full-thickness resection is achieved without exposing the gastric cavity, thus reducing the subsequent recurrence of peritoneal tumor seeding. Many studies have shown the feasibility of this procedure. However, this procedure is only for lesions less than 3 cm, due to its limitations in retracting the lesion transorally [52, 53].

### 4.8. Combination of Laparoscopic and Endoscopic Approaches to Neoplasia with Nonexposure Technique

CLEAN-NET was first developed by Inoue et al. [55] in 2012, based on a method called “suture first, cut later”. This method permits a full-thickness resection without exposing the gastric lumen to the peritoneal space, thus avoiding peritoneal seeding [54]. The standard procedure includes indicating and injecting a solution into the submucosal layer around the lesion endoscopically, dissecting the seromuscular layer laparoscopically (leaving the mucosa intact), pulling the lesion outwards by sutures placed at the lesion laparoscopically, and achieving complete resection by closing the defect with a laparoscopic stapling device [56]. Its advantages over the NEWS technique lies in the larger size that can be resected using the CLEAN-NET technique (>4 cm). The tumor located on the posterior wall can be very challenging when removed endoscopically [56]. Moreover, this technique is difficult for large intraluminal protrusions, which make it difficult to place the stapling device. Secondly, the accuracy of mucosal resection is lower when compared to the NEWS technique, since the incision line is determined from the serosal side [57].

## 5. Follow-up

The guidelines of the NCCN recommended an abdominal and pelvic CT scan every 3–6 mo for 3–5 years and an annual postoperative follow-up, whereas for very small tumors (<2 cm), less frequent observation is acceptable. Incompletely resected tumors or the presence of metastasis mandate an abdominal and pelvic CT scan every 3–6 mo. CT or MRI may be used to determine the progression, while PET/CT can be considered when CT or MRI is ambiguous. To assess unresectable, recurrent, and metastatic disease, as well as the response to preoperative imatinib treatment, an abdominal and pelvic CT scan or MRI is indicated every 8–12 weeks.

## 6. Conclusion

With an improvement in the knowledge of the pathogenesis of GISTs, accurate diagnosis and treatment can be achieved. Endoscopic treatment of GISTs for the upper GI is feasible and safe, with a relatively acceptable rate of complications. Major complications like perforations should best be avoided. Meanwhile, if perforation occurs, secondary complications like intraperitoneal infection and emphysema should be prevented. Nowadays, newly developed endoscopic procedures are challenging conservative surgery. Although surgery remains the standard therapy for primary and localized GISTs [58], many studies have proved that a minimally invasive treatment by endoscopy is feasible and safe in upper GISTs with sizes of <5 cm. Surgery is associated with higher morbidities and mortalities, and it impairs a patient's quality of life afterwards. A study by Yin et al. [59] proposed 3 different minimally invasive procedures for GISTs ≤ 5 cm. It showed that the ESD procedure had a significant difference in mean operative time and intraoperative bleeding when compared to laparoscopic resection (LAP) and LECS procedure (*P* < 0.001). The mean operative times of ESD, LECS, and LAP were 32.96 ± 11.76 min, 65.33 ± 20.57 min, and 81.67 ± 22.49 min, respectively, while the volumes of mean intraoperative blood loss were 6.98 ± 3.58 ml, 20.00 ± 13.50 ml, and 19.50 ± 11.55 ml, respectively. Thus, the endoscopic approach definitely has some benefits over laparoscopic or open surgery to some limit. The treatment of upper GIST by the endoscopic method is still controversial. A team approach involving an endoscopist, pathologist, radiologist, oncologist, and surgeon is the optimum in the management of a GIST in order to achieve R0 complete resection with minimal complications. However, more studies with relatively long-term outcomes should be carried out and conclusions about the oncological feasibility of endoscopic treatments should be made.

## Figures and Tables

**Table 1:** Relevant outcomes of the endoscopic enucleation procedure for gastrointestinal subepithelial tumors.

Study	*n*, GIST^1^	Method	Mean tumor size (mm)	Mean procedure time (min)	Complete resection rate (%)	Complication (%)	Mean follow-up (mo), recurrence
Sun et al. [26] (2007)	29, 29	EBL	8.0 (body)	—	96.0	3.4	41, 1
			9.0 (fundus)				
			11.0 (cardia)				
Nan et al. [28] (2014)	192, 177	EBL	8.0	—	100	1.0	—
He et al. [30] (2013)	31, 31	ESD	27.0	70.2	100	29.0	14.3, 0
Meng et al. [32] (2016)	68, 49	ESD	25.8	99.3^2^	98.5	11.8	12.9, 0
	47, 31	LWR	37.1	125.2^2^	100	23.4	11.1, 0
Liu et al. [33] (2012)	31, 16	EMD	22.1	76.8	97	12.9	17.7, 0
Ye et al. [35] (2014)	85, 19	STER	19.2	57.2	100	4.7	8.0, 0
Gong et al. [36] (2012)	12, 7	ESTD	19.5	48.3	83.3	16.7	—
Chen et al. [37] (2015)	180, 28	STER	26.0 (median)	45 (median)	90.6	8.3	36 (median), 0
Li et al. [38] (2015)	32, 11	STER	23.0	51.8	100	43.8	28.0, 0

^1^Total number of pathologically diagnosed GIST. ^2^Mean procedure time for GIST with a size of 20–50 mm. EBL = endoscopic band ligation; ESD = endoscopic submucosal dissection; LWR = laparoscopic wedge resection; EMD = endoscopic muscularis dissection; STER = submucosal tunneling endoscopic resection; ESTD = endoscopic submucosal tunnel dissection.

**Table 2:** Relevant outcomes of the endoscopic full-thickness resection and endoscopic-laparoscopic cooperative procedure for gastrointestinal subepithelial tumors.

Study	*n*, GIST^1^	Method	Mean tumor size (mm)	Mean procedure time (min)	Complete resection rate (%)	Complication (%)	Mean follow-up (mo), recurrence
Zhou et al. [44] (2011)	26, 16	EFTR	28.0	105.0	100	0	8.0, 0
Feng et al. [45] (2014)	48, 43	EFTR	15.9	59.7	100	1.0	6.0–24 (range), 0
Kappelle et al. [48] (2017)	13, 2	EFTR^2^	11.0	—	84.6	38.5	3.0–6.0 (range), 0
Ye et al. [46] (2014)	51, 30	EFTR	24.0	52.0	98.0	0	22.4, 0
Hiki et al. [49] (2008)	7, 7	LECS	46.0	169.0	100	0	—
Namikawa and Hanazaki [50] (2015)	8, 8	LECS	31.0	213.0	100	0	—
Mitsui et al. [52] (2011)	6, 5	NEWS	34.8	273.5	100	0	8, 0
Goto et al. [53] (2016)	20, —^3^	NEWS	—^3^	213.5	100	5.0	10.1, 0
Nabeshima et al. [54] (2015)	2, 2	CLEAN-NET	37.5	165.0^4^	100	0	—
Hajer et al. [56] (2018)	10, 4	NEWS CLEAN-NET	32.7	99	100	20	—
	2, 2		37.5	150	100	0	

^1^Total number of pathologically diagnosed GIST. ^2^EFTR using a new flat-based over-the-scope clip. ^3^Data unavailable due to limited access. ^4^One case underwent CLEAN-NET and cholecystectomy procedure. EFTR = endoscopic full-thickness resection; LECS = laparoscopic endoscopic cooperative surgery; NEWS = nonexposed endoscopic wall-inversion surgery; CLEAN-NET = endoscopic approaches to neoplasia with nonexposed technique.
